# A Wireless MEMS-Based Inclinometer Sensor Node for Structural Health Monitoring

**DOI:** 10.3390/s131216090

**Published:** 2013-11-26

**Authors:** Dae Woong Ha, Hyo Seon Park, Se Woon Choi, Yousok Kim

**Affiliations:** 1 Department of Architectural Engineering, Yonsei University, 134 Shinchon-dong, Seoul 110-732, Korea; E-Mails: dwha@yonsei.ac.kr (D.W.H); hspark@yonsei.ac.kr (H.S.P.); 2 Center for Structural Health Care Technology in Buildings, Yonsei University, 134 Shinchon-dong, Seoul 110-732, Korea; E-Mail: watercloud@yonsei.ac.kr

**Keywords:** structural health monitoring, inclinometer, wireless sensor network, sensor network management

## Abstract

This paper proposes a wireless inclinometer sensor node for structural health monitoring (SHM) that can be applied to civil engineering and building structures subjected to various loadings. The inclinometer used in this study employs a method for calculating the tilt based on the difference between the static acceleration and the acceleration due to gravity, using a micro-electro-mechanical system (MEMS)-based accelerometer. A wireless sensor node was developed through which tilt measurement data are wirelessly transmitted to a monitoring server. This node consists of a slave node that uses a short-distance wireless communication system (RF 2.4 GHz) and a master node that uses a long-distance telecommunication system (code division multiple access—CDMA). The communication distance limitation, which is recognized as an important issue in wireless monitoring systems, has been resolved via these two wireless communication components. The reliability of the proposed wireless inclinometer sensor node was verified experimentally by comparing the values measured by the inclinometer and subsequently transferred to the monitoring server via wired and wireless transfer methods to permit a performance evaluation of the wireless communication sensor nodes. The experimental results indicated that the two systems (wired and wireless transfer systems) yielded almost identical values at a tilt angle greater than 1°, and a uniform difference was observed at a tilt angle less than 0.42° (approximately 0.0032° corresponding to 0.76% of the tilt angle, 0.42°) regardless of the tilt size. This result was deemed to be within the allowable range of measurement error in SHM. Thus, the wireless transfer system proposed in this study was experimentally verified for practical application in a structural health monitoring system.

## 1. Introduction

Performance degradation of civil engineering and building structures due to various loads and aging over their life cycles is an important evaluation factor in terms of the safety of both the structures and their users. To this end, studies on the application of structural health monitoring (SHM) techniques to civil and building structures have been conducted using a variety of sensors, and the range of applications to various types of structures has increased [1–3].

The major measurement responses used in evaluating the integrity of these structures are acceleration, displacement, and strain. Changes in the dynamic characteristics of the structures can be estimated using acceleration measurement data to evaluate the damage that has occurred in the structures [4,5]. Although the global damage to the structures can be estimated using accelerometers; however, it is difficult to determine the location and extent of the damage, such as local damage to a particular structural member. The commonly used displacement sensors measure the relative displacement within the structure [6], and thus, space is required to secure the reference point, which renders application difficult because of various limitations at the actual site. On the other hand, strain-type sensors yield good results in terms of accuracy and applicability in measuring damage to local structural members and are applicable in practice to many field cases [7–9]; however, a large number of sensors need to be installed to evaluate the integrity of all structural members or to obtain reliable results for a whole structure.

Together with the existing structural response indices that are primarily adopted in the SHM area (*i.e.*, acceleration, displacement and strain), the occurrence of tilt during structural deformation provides information that is useful in evaluating the vertical deflection via the angle of rotation in the case of horizontal members and the drift in the case of vertical members. As such, an inclinometer can evaluate the deformation of members using the angle of rotation, such that tilt measurements can be used as primary measures in evaluating the safety of individual structural members and entire structures.

Inclinometer sensors for tilt measurements have been widely applied in many industrial applications. The automotive, electronics, and aviation industries are among the major areas of application [10–12]. The concept behind an inclinometer is that it performs measurements of various responses generated by pendulum behavior caused by gravity. The pendulum types are largely represented by the categories of solid mass, liquid, and gas [13,14], and resistive, capacitive, inductive, magnetic, fiber optic, and optical methods are used to measure the response of the pendulum with respect to gravity [10,15]. Properties such as small size, low weight, and accuracy are necessary conditions for the application of inclinometers to civil engineering and building structures, and inclinometer sensors that meet these conditions have been developed. Among the inclinometer sensors developed to date, micro-electro-mechanical system (MEMS)-based inclinometers are highly applicable to SHM due to their characteristics (e.g., their durability, compactness, wireless nature, processor type, storage capability, miniaturizability, multiple component structure, immunity from electronic noise), and many successful applications have been reported. The MEMS-based inclinometers that have been developed to date are based on a piezoresistor structure [13], an electrolytic tilt sensor [16,17], or an optical inclination sensor [10], depending on the measurement method used. In particular, a MEMS-based inclinometer sensor that uses an accelerometer has been proposed, along with a MEMS-based accelerometer [18,19], the principle of which is based on measuring the changes in the tilt caused by the acceleration due to gravity.

In addition to research and development for sensors in the SHM area, another important issue is the development and application of wireless communication methods. The importance of wireless communication lies in its ability to overcome the many limitations [20,21] that confront the construction of wired monitoring systems in both existing buildings and buildings under construction and economic inefficiency in terms of installation, maintenance, and management of existing wired monitoring systems. The importance of and need for the development of a wireless monitoring system can be verified via the practical applications of existing wireless monitoring systems [3,22]. Various wireless technologies with a variety of sensors have been developed and applied since Straser first reported the practical application of a wireless communication method [23]. However, many technical problems (e.g., power consumption; time synchronization; multi-scale network topologies—*i.e.*, scalability; decentralized data processing; and power-efficient data-driven usage strategies) arise that must be solved before a wireless monitoring system can be applied to various structures on a long-term basis. The application of such a wireless monitoring system is essential for efficient SHM implementation for such structures, which have become increasingly large, tall, and complex. Wireless sensors have been developed and applied in various experiments, and the aforementioned problems have been addressed through an extensive series of experimental studies. However, despite the development of various wireless sensors [24–26] and numerous applications, only a few developmental studies have been conducted on wireless systems for inclinometers, particularly for structures.

In this study, a monitoring system that is suitable for structures was developed using an inclinometer unit with an accelerometer based on MEMS technology. In addition, a wireless inclinometer sensor node was developed to wirelessly transfer the measurement data from an inclinometer. Moreover, a wireless system has been developed that overcomes the limitation of wireless communication distance by combining a short-range wireless communication device located at the measurement site with a long-distance wireless communication module that enables remote monitoring and management of the measurement data from the measurement site. This advancement solves the problem of the transfer distance limitation, which has long been recognized as a problem in existing wireless monitoring systems. Finally, a tilt measurement experiment was conducted to verify the reliability and usability of the wireless MEMS-based inclinometer sensor node proposed in this study. The objective of this experiment was to verify the reliability of the wireless transfer method by comparing the measurement results transmitted to the server by both wired and wireless communication.

## 2. MEMS-Based Inclinometer Sensor

### 2.1. Inclinometer Principles

Various sensors for MEMS-based inclinometers have been developed using different measurement methods. In this study, an inclinometer that uses an accelerometer based on MEMS technology was adopted for the SHM. The MEMS-based accelerometer responds sensitively to gravity; thus, a MEMS-based accelerometer sensor in a stationary state measures both the static acceleration and the acceleration due to gravity. In this case, a certain angle is generated between the static acceleration and the acceleration due to gravity. This angle corresponds to the slope of the sensor or the so-called tilt.

**Figure 1.**
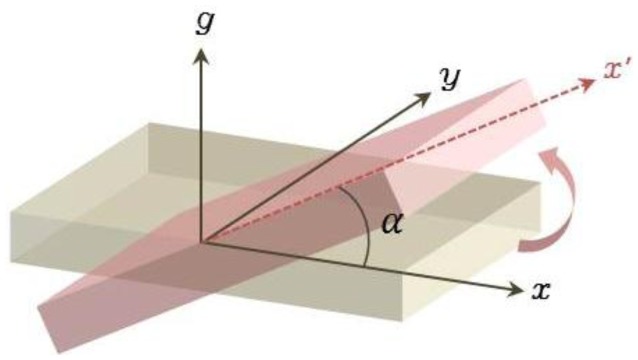
X-axis tilt assignment relative to the ground.

Figure 1 presents the acceleration along the x-axis (*a_x_*) and the acceleration due to gravity (*g*) generated by changes in the location of the sensor. The relationship between *a_x_* and *g* is expressed in Equations (1) and (2), where α denotes the slope of the sensor:

(1) $$a_{x}=g\cdot \sin\alpha$$

(2) $$\alpha =\sin^{-1}(a_{x}/g)$$

### 2.2. Composition and Manufacture of an Inclinometer

An SCA103T chip developed by Murata Electronics Oy (Vantaa, Finland) [27] was used as the sensing element in the manufacture of the MEMS-based inclinometer. The SCA103T chip exhibits two measurable ranges in a single-axis inclinometer, namely, ±15° and ±30°, with a precision of 0.001°, and thus, it is considered suitable for civil engineering and construction applications. The SCA103T chip is also considered suitable under harsh external conditions, such as those occurring in structural construction fields, because of its durability with respect to temperature and shock. In addition, this chip is only slightly affected by electrical interference and can reduce disturbance by other sensors.

**Figure 2.**
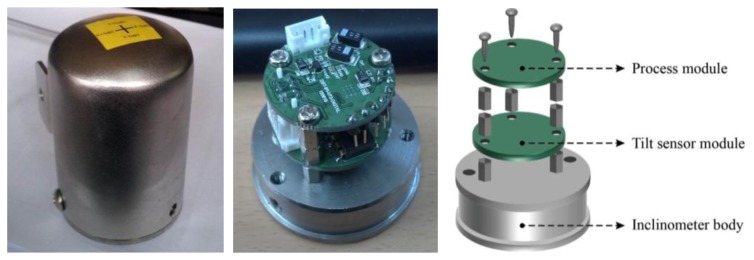
MEMS-based inclinometer.

Sensor durability is an essential factor in SHM applications, considering the notably harsh environments at construction sites and over the long term. Miniaturization and light weight design are also necessary factors for increasing the applicability at an actual site. Therefore, the external component of the inclinometer sensor used in this study is constructed of aluminum, which is lightweight and durable (Figure 2), thereby preventing physical damage to the sensor by the harsh external environment. In addition, modules consisting of inclinometer sensors are classified into tilt sensor modules and process modules, thereby affording flexibility in the module arrangement. The modules are manufactured as shown in Figure 2, and the two modules are divided into upper and lower layers to produce a smaller contact area of the inclinometer installed on a structure. The diameter and the height of inclinometer are 50 mm and 78 mm, respectively, and its total weight is 440 g.

**Figure 3.**
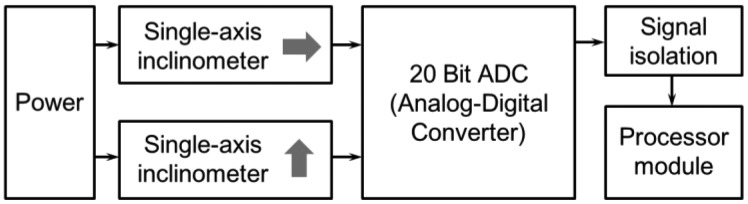
Block diagram of the tilt sensor module.

**Figure 4.**
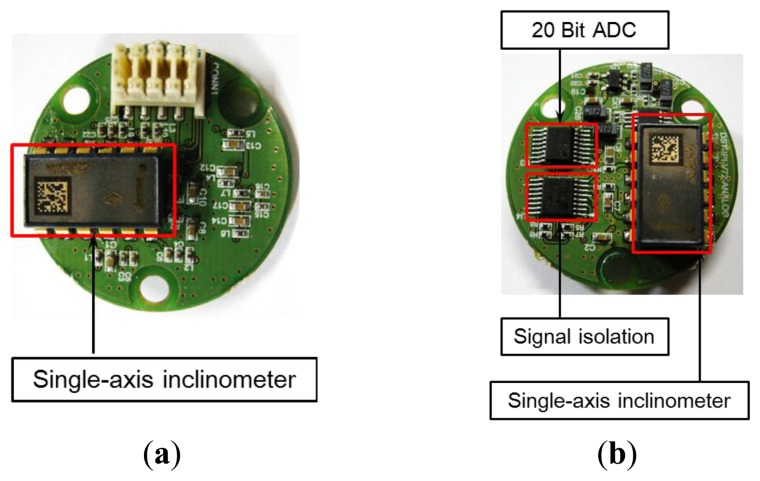
Tilt sensor module. (**a**) Upper surface; (**b**) Lower surface.

The tilt sensor module measures the slope, and its composition is shown in Figure 3. Two single-axis inclinometers (SCA103T), which are needed to measure tilts, are placed one on top of the other orthogonally at the upper and lower surfaces, thereby measuring the tilt in the two horizontal x- and y-axis; directions. The tilts measured by each single-axis inclinometer are output as analog values. The analog values are converted to 20-bit digital values via an ADC converter. The digital signal is transmitted to the processor module located on the upper surface. Signal isolation is performed because the output values from the single-axis inclinometer experience interference from the transmitted digital signal noise. Figure 4 presents the upper and lower surfaces of the tilt sensor module inside the sensor.

**Figure 5.**
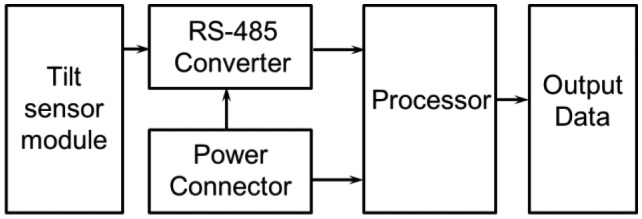
Block diagram of the processor module.

**Figure 6.**
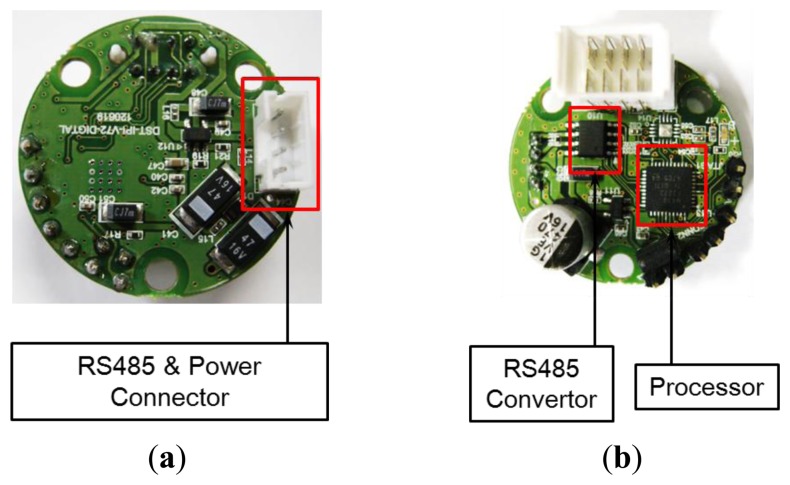
Processor module. (**a**) Front side; (**b**) Back side.

The processor module (Figure 5) is responsible for controlling the output value of the inclinometer sensor. The digital signal output from the tilt sensor module is converted to an RS-485 signal by an RS-485 converter. The converted signal is transferred to a slave node connected by a wire, the length of which depends on the condition of the site where the inclinometer is installed and can be extended to 1,200 m at a communication speed of 100 kbps. However, it is desirable to shorten its length to minimize the problems associated with establishing and maintaining a wired connection. Figure 6 presents the upper and lower surfaces of the processor module. The processor chip on the back side plays a major role in controlling the overall operation of the processor module.

## 3. Composition of the Wireless Inclinometer Sensor Node

The wireless MEMS-based inclinometer monitoring system developed here consists of an inclinometer sensor unit (described in Section 2), a sensor node, and a monitoring server. A wireless sensor node was developed to wirelessly transfer the data measured by the MEMS-based inclinometer sensor. The wireless sensor node is equipped with a multiport interface and is simultaneously connected to a number of inclinometer sensors to take measurements and remotely transmit the measured results.

**Figure 7.**
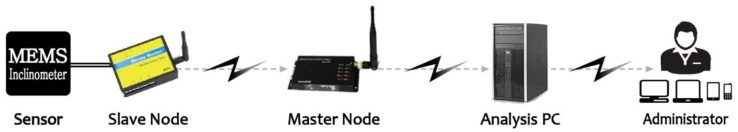
Wireless MEMS inclinometer sensor system.

The wireless sensor node refers to both the slave and master nodes, the dimensions and weight of which are 98 × 69 × 33 mm and 300 g, respectively. The slave node is responsible for transmitting the data between the MEMS-based inclinometer and the master node. In this case, the inclinometer and the slave node are connected by a wire, and the slave node is responsible for receiving the output data from the inclinometer sensor unit and wirelessly transmitting the data to the master node located a short distance away (Figure 7). In the slave node, four channels can be configured, and the same number of MEMS-based inclinometer sensors as the number of channels configured can be connected simultaneously in current state; a maximum of 24 channel can be configured in future development.

**Figure 8.**
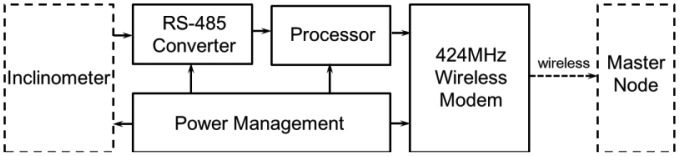
Slave node diagram.

**Figure 9.**
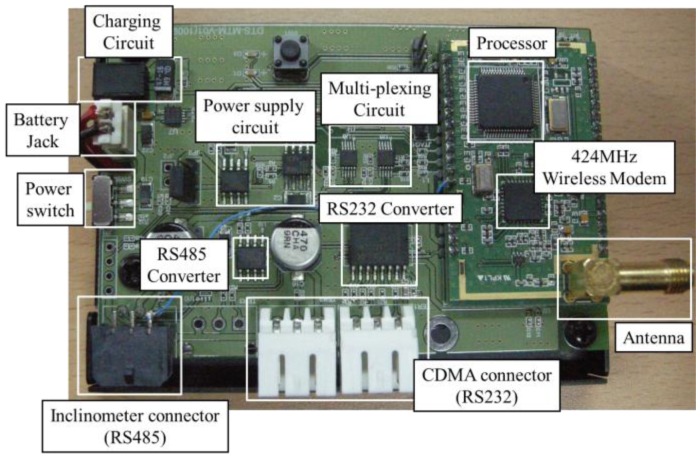
Slave node.

A 2.4-GHz radio frequency (RF) is used as a short-range communication method between the slave and master nodes. The RF wave mode is used for distances between 30 and 120 m. RF waves are suitable for building structures that consist of many structural and temporary members because only slight refraction and diffusion by obstacles occur with these waves. RF waves consume less power during transmission than other communication methods and are therefore well suited to long-term measurement and short-range communication. A CC1020 chip [28] is used as the wireless data conversion device because this device can effectively convert an RF signal with low power. This type of chip is used in many industrial fields. Figure 8 presents a diagram of the interior structure of the slave node. In the slave node, the RS-485 signal transmitted from the MEMS-based inclinometer sensor is passed through the processor in the slave node and is demodulated into an RF signal for wireless communication via the RF integrated circuit (IC). Next, this signal is transmitted wirelessly to the master node. The board of the wireless slave node is shown in Figure 9, which illustrates each component for communication, processing, and power supply.

Once the master node receives the data transmitted wirelessly from the slave node, it transmits the received data to the monitoring server (PC) or to another master node in a remote location. The code division multiple access (CDMA, [29]) method is used as a long-distance wireless communication method. CDMA is a communication method for mobile devices that has been adopted in Korea, and therefore, long-distance communication of the type allowed with mobile phones is possible as long as the base station for CDMA communication can cover the area. This communication method has the advantage of minimizing the effects of a number of obstacles due to the field characteristics of the monitored structures.

**Figure 10.**
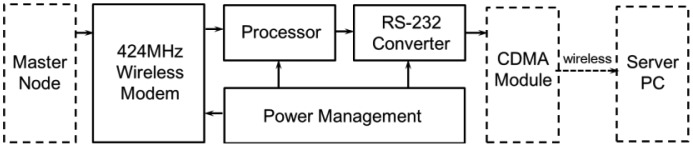
Master node diagram.

**Figure 11.**
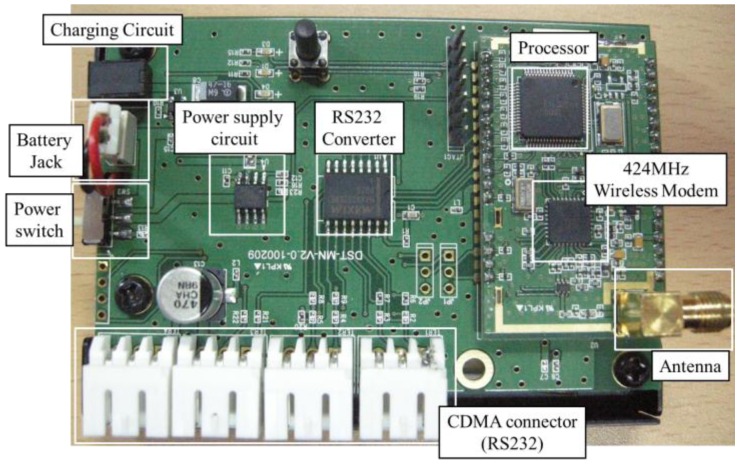
Master node.

Figure 10 presents the interior structure of the master node. The data processing method is similar to that of the slave node, whereas the master node uses an RS-232 method for communication with the CDMA module. Each component in the master node is illustrated in Figure 11.

The wireless sensor system requires a general power supply for the sensor and sensor node. The currently developed power supply has a limited life because it is often difficult to obtain access to the system to replace the battery and because it is often difficult to supply continuous power to the system, depending the characteristics of the structure in which the wireless sensor is installed. The power supply device used in this study is a lithium polymer battery [30] that is connected to the slave node and provides both the slave node and the inclinometers with electric power. The life of a fully charged battery of this type is approximately 20 h at a discharge rate of 4,400 mA/h. In this study, the sensor and sensor nodes were operated continuously during testing, and no low-power technology, such as switching between sleep and active modes, was employed. However, implementation of power-saving strategies [24] that can control the operating time depending on the scheduled measurement interval and therefore minimize power consumption is planned for future development. The batteries can be recharged as often as possible once accessibility is ensured. Nonetheless, the long-term measurement capability of the system is limited because most construction locations are not readily accessible. Recently, various charging methods have been studied, including a solar charging system, a chemical power device, and a system that uses the vibrational energy of a structure; however, none of these methods has yet been applied in the field. Therefore, further study of high-density energy storage technology and energy harvesting for wireless sensor nodes, which is particularly important in the monitoring of historical buildings, is needed [31–34].

## 4. Experiment

### 4.1. Test Setup

**Figure 12.**
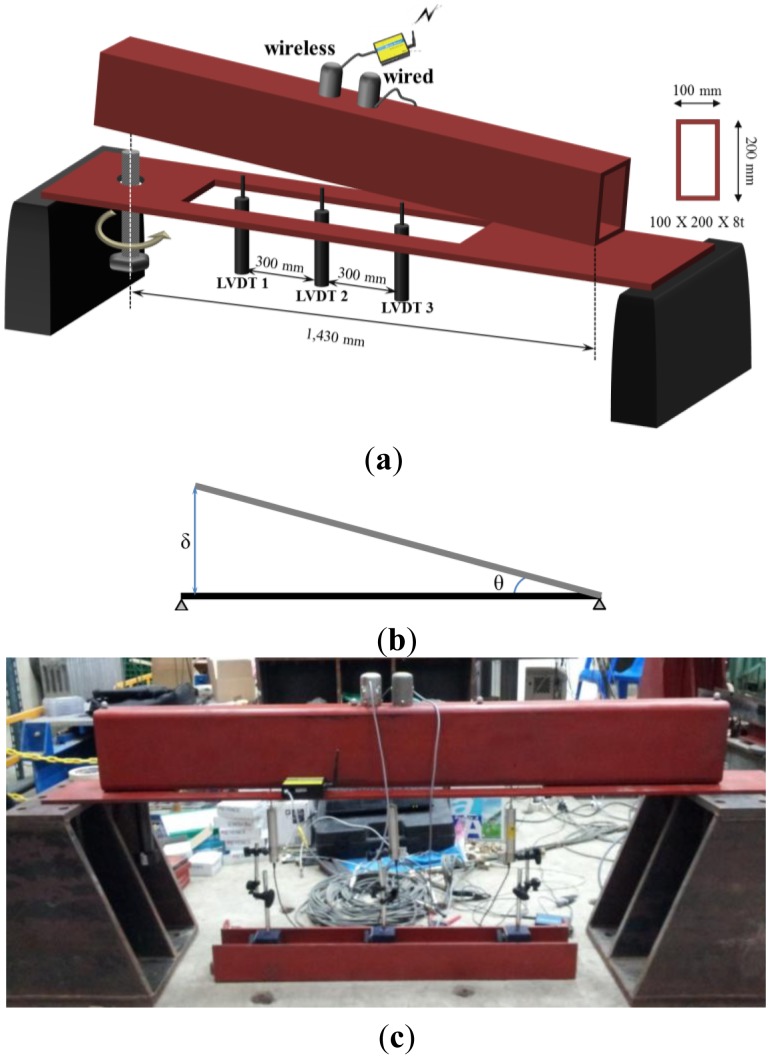
Test setup and instrumentation. (**a**) Schematic view of the test setup; (**b**) θ calculated from LVDTs; (**c**) Front view of the specimen.

To verify the reliability of the wireless MEMS-based inclinometer sensor node developed in this study, the specimen shown in Figure 12 was manufactured to conduct a tilt measurement experiment. The main objective of this experiment was to verify the applicability of the system by comparing the measurements obtained with the wired inclinometer with the output values of the wireless transmission from the wireless sensor node. This verification was accomplished using two sets of the same type of MEMS-based inclinometers described in Section 2, installed on a specimen using glue, as shown in Figure 12. The results obtained by the two different communication methods (wired and wireless) were compared. In this case, wired communication refers to wired transmission from the inclinometer sensor to the server PC, and wireless communication refers to the transmissions among the inclinometer, slave node, master node, and server PC.

An M36 screw was installed at one end of the test specimen to generate tilt in the upper member, as shown in Figure 12. A vertical displacement was generated at one end by turning the screw. In this case, the vertical displacement (δ in Figure 12b) generated by a one-pitch revolution of the M36 screw was 4 mm, which was how the slope was adjusted. The upper member in which the tilt was generated consisted of a square steel member with dimensions of 100 × 200 × 8 (mm). Linear variable displacement transducers (LVDT) were installed beneath the upper member to measure the tilt of the member, which was set based on the vertical displacement values measured by LVDTs at three measurement locations (Figure 12b). The average of the tilt values calculated from the three LVDT measurements was used as a reference.

Experiments were conducted for two sets of slope increments. In the first experiment (Case 1), the increment Δ*θ* was set to approximately 0.04°, and 11 steps in total were performed. In the second experiment (Case 2), the increment Δ*θ* was set to 1°, and measurements from 0° to 5° were performed in a total of six steps. These two experiments with the incremental changes in slope described were conducted to verify the accuracy of the wireless communication output values relative to the measured values.

### 4.2. Test Results

**Figure 13.**
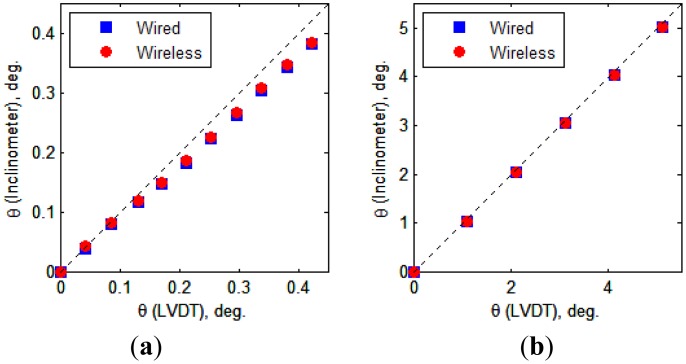
Relationship between inclinations calculated from LVDTs measurements and measured by inclinometers. (**a**) Case 1 (0–0.38°); (**b**) Case 2 (0–5°).

**Figure 14.**
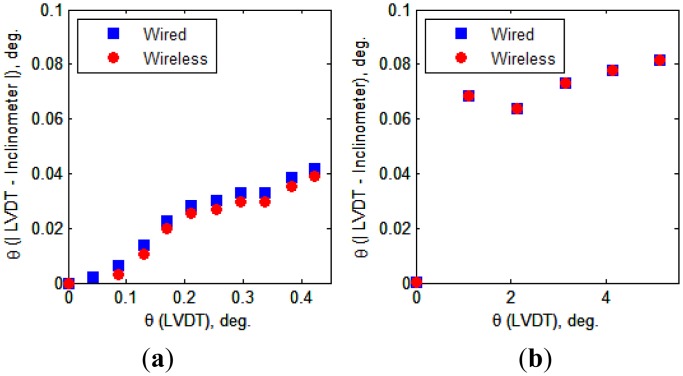
Differences in inclinations calculated from LVDT measurements and measured by inclinometers. (**a**) Case 1 (0–0.38°); (**b**) Case 2 (0–5°).

Figure 13 presents the tilt values obtained from the experiments. The tilt values calculated from the LVDT measurements are compared with the inclinometer values obtained from the wired and wireless transmissions. For the case in which the inclination ranged from 0 to 0.38° (Case 1), the tilt values measured by the inclinometers are slightly smaller than those calculated from the LVDT measurements. For the case in which the inclination ranged from 0 to 5° (Case 2), the inclinometer values are nearly the same as those calculated from the LVDT measurements. With respect to the different tilt ranges of the two cases, the differences in the measured values between the two devices (*i.e.*, LVDTs and inclinometers) are illustrated in Figure 14, which shows that the absolute differences increase as the tilt value increases. The maximum difference between the two measurement methods (*i.e.*, LVDTs *vs.* inclinometers) was 0.082°, which is considered within the allowable measurement error range for the test.

**Figure 15.**
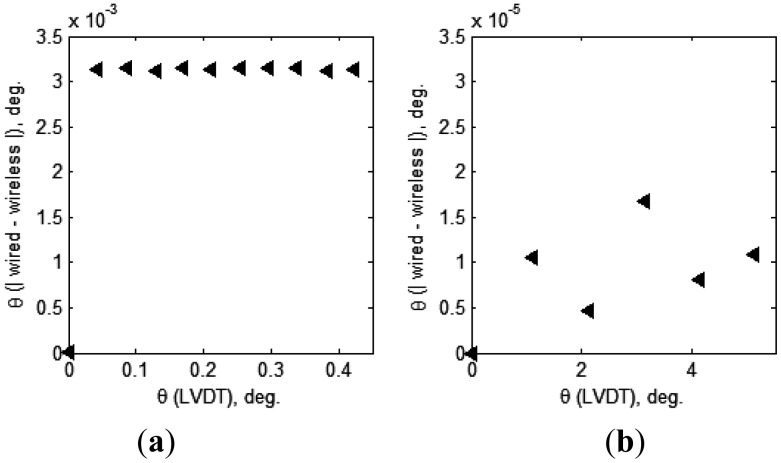
Differences in inclinations measured by wired and wireless inclinometers. (**a**) Case 1 (0–0.38°); (**b**) Case 2 (0–5°).

Figure 15 and Table 1 present the differences between the tilt values obtained from the wired and wireless inclinometers that transmit measurements via a cable and the proposed wireless sensor node, respectively. In Case 1, nearly constant differences (approximately 0.0036°) were observed between the tilt values obtained by the two transmission methods (wired and wireless), regardless of the tilt value. However, Case 2 did not produce any consistent results because the inclination increases and the differences calculated in Case 2 were considerably smaller than those in Case 1 (note that the scales of the vertical axes of the two plots in Figures 15a,b are different).

The results obtained indicate that when the tilt exceeds 1°, the differences between the results obtained using the wired and wireless communication methods are small, whereas the difference is larger (approximately 0.0032°) when the tilt is less than 0.42°. In other words, larger differences between the two transmission methods are observed for smaller tilts. Nonetheless, the maximum error value measured was 0.0032° corresponding to 0.76% of the tilt angle, 0.42°, which is an error of negligible magnitude in SHM applications for civil engineering and building structures. Therefore, the accuracy of the wireless inclinometer node proposed in this study was considered to have been successfully verified by the experiment results. Thus, this wireless inclinometer node could replace existing wired methods without significant loss of measurement accuracy.

## 5. Conclusions

In this study, an inclinometer unit suitable for use in SHM of civil engineering and building structures was manufactured using an inclinometer sensor based on a MEMS-based accelerometer, and a wireless inclinometer sensor node was developed for use in an efficient monitoring system via wireless transmission of tilt measurements. A tilt measurement experiment was conducted with a wired inclinometer to verify the accuracy of the wireless transmission system and the wireless inclinometer sensor node developed in this study. The results obtained from this study are summarized as follows:

1. By separating the sensor and processor modules in the manufacture of the inclinometer sensor unit, the flexibility of the unit, in terms of module placement and manufacture, was increased. The two modules were located on the upper and lower layers of the unit, thereby reducing the contact area of the inclinometer sensor and increasing the practical applicability of the system in actual structures.
2. The wireless sensor node for wireless communication consisted of a slave node and master node. Using these nodes, a system was implemented to wirelessly transmit tilt data measured using an inclinometer sensor to a monitoring server at a remote location. The slave node received the data from a number of inclinometers simultaneously through a wire and a multichannel interface and transmitted the received data to the master node via a wireless communication method (RF 2.4 GHz). The master node transmitted the measurement data received from the slave node to a remotely located monitoring server using CDMA transmission. This wireless monitoring system was able to overcome the transmission distance limitation of the existing wireless communication method.
3. To verify the transmission accuracy of the wireless communication through sensor nodes developed in this study, two sets of MEMS-based inclinometer sensors were installed, and the measurement data obtained in both wired and wireless modes were compared. Wired communication refers to wired transmission from the inclinometer sensor to the server PC, whereas wireless communication refers to transmission among the inclinometer, slave node, master node, and server PC. The results of the comparison of the measurement data indicated that the two sets of measurements were nearly the same when the tilt was greater than 1°. The maximum difference of 0.0032° was observed when the tilt was less than 0.4°. The differences in the measurements obtained using the two transmission methods were considered negligible for SHM purposes. Therefore, our experiment verified that the wireless inclinometer sensor node proposed in this paper could successfully replace a wired inclinometer.
4. The accuracy of the wireless sensor node was verified in laboratory tests in which the environmental conditions could be conveniently controlled. A more general and universal evaluation of the applicability of the wireless sensor node will require verification using real structures with many obstructions that hinder wireless communication.

## Figures and Tables

**Table 1.** Differences in inclinations measured by wired and wireless inclinometers.

**Tilt Values from LVDT (deg.)**	**Wireless–Wired (×10^−3^deg.)**	**(Wireless–Wired) /LVDT (%)**
0.04	3.14	7.49
0.09	3.16	3.66
0.13	3.12	2.39
0.17	3.15	1.85
0.21	3.13	1.48
0.25	3.14	1.24
0.30	3.14	1.06
0.34	3.15	0.93
0.38	3.12	0.82
0.42	3.13	0.74
1.11	0.01	0.00
2.12	0.00	0.00
3.13	−0.02	0.00
4.12	0.01	0.00
5.11	0.01	0.00
